# LncRNA MIR503HG serves as a diagnostic biomarker in patients with sepsis and regulates sepsis‐induced inflammation and cardiac dysfunction

**DOI:** 10.1002/ccs3.70078

**Published:** 2026-05-26

**Authors:** Pingping Qi, Minghe Jiang, Minsheng Wu, Shengkui Chen, Qile Ye, Rong Tang

**Affiliations:** ^1^ Blood Transfusion Department The Second Affiliated Hospital of Guangxi Medical University Nanning China; ^2^ Department of Intensive Care Unit Ruikang Hospital Affiliated to Guangxi University of Chinese Medicine Nanning China; ^3^ Emergency Department Ruikang Hospital Affiliated to Guangxi University of Chinese Medicine Nanning China; ^4^ Department of Critical Care Medicine The Second Affiliated Hospital of Harbin Medical University Harbin China

**Keywords:** BDNF, inflammation, miR‐497‐5p, MIR503HG, sepsis

## Abstract

Sepsis often has a dysregulated inflammatory response and is accompanied by cardiac dysfunction. This study aimed to explore the mechanism of long non‐coding RNA MIR503HG (MIR503HG) in regulating sepsis and the inflammatory responses, and sepsis‐induced cardiac dysfunction (SICD). 102 sepsis patients were divided into an SICD group (*n* = 31) and a non‐SICD group (*n* = 71). A cecal ligation and puncture (CLP) sepsis rat model was constructed. Quantitative reverse transcription polymerase chain reaction (RT‐qPCR) was used to evaluate the target gene expression. Enzyme‐linked immunosorbent assay was used to measure the levels of myocardial damage markers and inflammatory factors. RNA immunoprecipitation and Dual luciferase assay were used to determine the targeting relationship. Kyoto Encyclopedia of Genes and Genomes analysis was used to predict signaling pathways of target genes. MIR503HG expression was significantly down‐regulated in sepsis patients and SICD patients, and it has good diagnostic value for these two types of diseases. Its expression was significantly negatively correlated with myocardial injury markers (cardiac troponin I [cTnI], creatine kinase‐MB [CK‐MB]), cardiac function indicators (left ventricular ejection fraction), inflammatory factors (interleukin‐6, tumor necrosis factor‐alpha), and disease severity scores (Sequential Organ Failure Assessment, Acute Physiology and Chronic Health Evaluation II). Furthermore, it exerted a protective effect in sepsis: MIR503HG overexpression could effectively alleviate SICD and mitigate the inflammatory response, as evidenced by decreased left ventricular end‐diastolic pressure, increased left ventricular systolic pressure, reduced levels of CK‐MB and cTnI, as well as restored myocardial systolic/diastolic capacity (maximal rate of left ventricular pressure rise/fall [±dP/dt]). Mechanistically, MIR503HG functions as a molecular sponge that sequesters microRNA‐497‐5p (miR‐497‐5p), thereby lifting brain‐derived neurotrophic factor (BDNF). Delivery of miR‐497‐5p‐agomiR partially offset the cardioprotective and anti‐inflammatory effects of MIR503HG, whereas overexpression of BDNF partially restored them. MIR503HG relieved sepsis and reduced cardiac dysfunction and inflammatory response by regulating the miR‐497‐5p/BDNF axis.

## Graphical abstract

MIR503HG was downregulated in sepsis and sepsis‐induced cardiac dysfunction (SICD) and served as a diagnostic biomarker. Overexpression of MIR503HG attenuated myocardial injury and systemic inflammation in a septic rat model. Mechanistically, MIR503HG acted as a ceRNA by sponging miR‐497‐5p to upregulate BDNF, revealing a protective MIR503HG/miR‐497‐5p/BDNF axis in sepsis and SICD.

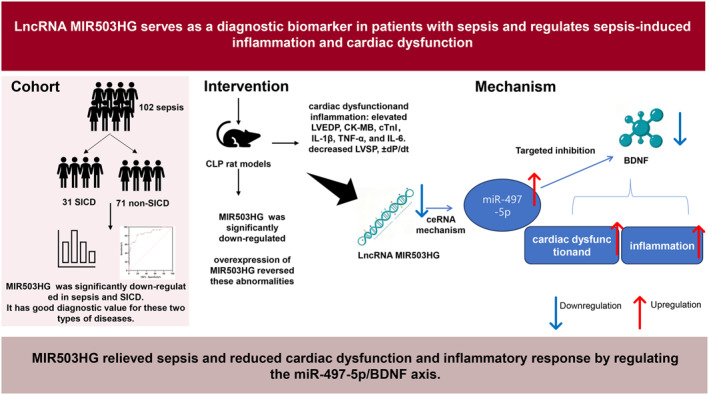

## 1 INTRODUCTION

The essence of sepsis is “life‐threatening organ dysfunction caused by the dysregulation of the host's response to infection”.^1^, ^2^ Dysregulated inflammatory response constitutes a central element in its pathogenesis.^3^, ^4^ Although the overall prognosis of patients with sepsis has improved to some extent in recent years, the mortality rate of patients with septic shock remains high.^5^, ^6^ Sepsis‐induced cardiac dysfunction (SICD), as an important type of multi‐organ dysfunction caused by sepsis, is a common serious complication during the progression of sepsis and is a significant risk factor for the death of sepsis patients.^7^, ^8^ Typical clinical manifestations include reduced ejection fraction, cardiac enlargement, and poor response to fluid resuscitation and catecholamine drugs.^9^ There is a lack of effective clinical treatments for sepsis and SICD.^10^, ^11^

Long non‐coding RNA (lncRNA) can participate in disease regulation through a variety of molecular mechanisms: as a competing endogenous RNAs (ceRNAs, molecular sponges), it competitively binds to microRNAs (miRNAs), blocks its regulatory effect on target messenger RNA (mRNA), participates in epigenetic regulation, and can also bind to mRNA at the post‐transcriptional level, affecting its shearing, stability and translation efficiency.^12^, ^13^ Previous studies have confirmed that LncRNA plays a functional role in inflammatory responses, vascular aging, rheumatoid arthritis, and oncology.^14^, ^15^, ^16^, ^17^ lncRNAs are critically involved in sepsis and the cardiac dysfunction and inflammatory response it triggers.^18^, ^19^ For instance, small nucleolar RNA host gene 8 (SNHG8) modulates sepsis‐induced inflammation and myocardial injury by acting as a sponge for miR‐34b‐5p,^20^ whereas the abundance of lncRNA HOTTIP is tightly linked to both disease severity and the development of SICD patients.^21^ In addition, MIR503HG can participate in tumors, inflammation, and other diseases through mechanisms such as ceRNA, protein binding, and epigenetic modification.^22^, ^23^ For example, inhibiting MIR503HG facilitates non‐small cell lung cancer metastasis.^24^ MIR503HG is involved in ventricular development, with its deletion inducing NOTCH pathway signaling changes and left ventricular noncompaction cardiomyopathy,^25^ whereas its downregulation promotes inflammation in abdominal aortic aneurysm cells.^26^ The ENCORI database indicates that MIR503HG may have a targeted binding relationship with microRNA‐497‐5p (miR‐497‐5p), and miR‐497‐5p has been proven to play a role in promoting pathological processes in various diseases.^27^, ^28^ In oxidized low‐density lipoprotein (ox‐LDL)‐challenged human umbilical vein endothelial cells, it amplifies endothelial dysfunction via activation of the p38/mitogen‐activated protein kinase (p38/MAPK) axis,^29^ whereas in LPS‐stimulated BEAS‐2B bronchial epithelial cells, its up‐regulation accelerates apoptosis and amplifies the inflammatory cascade.^30^ Conversely, downregulation of miR‐497‐5p protects BEAS‐2B from lipopolysaccharide (LPS)‐induced death and inflammation,^31^ and in the cecal ligation and puncture (CLP) rat model, blockade of miR‐497‐5p markedly attenuates LPS‐triggered cardiomyocyte injury.^32^ The intersection of TargetScan, miRDB, and ENCORI, three online databases, showed that miR‐497‐5p targets and regulates brain‐derived neurotrophic factor (BDNF). BDNF is a core member of the neurotrophic factor family (NTFs) and mainly regulates neuron survival, differentiation, synaptic plasticity, and nerve repair.^33^, ^34^ Recent studies have confirmed that BDNF is also widely expressed in cardiomyocytes, vascular endothelial cells, and cellular inflammation.^35^, ^36^ Notably, emodin enhances autophagy and prevents apoptosis in sepsis‐associated encephalopathy via activating the BDNF/tropomyosin receptor kinase B (BDNF/TrkB) pathway,^37^ and increased BDNF levels mitigate sepsis‐induced neuroinflammation.^38^ In summary, we speculate that MIR503HG may be involved in the regulation of sepsis and SICD and inflammatory response by regulating the miR‐497‐5p/BDNF axis.

Based on the above research evidence, this study first analyzed the MIR503HG, miR‐497‐5p, and BDNF expression in clinical samples, and constructed a cecal ligation and puncture (CLP) sepsis rat model to systematically explore the potential molecular mechanisms of the MIR503HG/miR‐497‐5p/BDNF regulatory axis in sepsis and sepsis‐induced myocardial functional damage and inflammatory response.

## 2 MATERIALS AND METHODS

### 2.1 Participants

This study was approved by the Ruikang Hospital Affiliated to Guangxi University of Chinese Medicine Ethics Committee, and all participants signed the informed consent form. This study was conducted in accordance with the principles outlined in the Declaration of Helsinki. 102 patients diagnosed with sepsis were recruited from Ruikang Hospital Affiliated to Guangxi University of Chinese Medicine. The inclusion of patients into the SICD group (*n* = 31) or non‐SICD group (*n* = 71) was determined by the following diagnostic criteria: serum cardiac troponin I [cTnI] concentration exceeding 0.01 μg/L, or left ventricular ejection fraction (LVEF) less than 50% as assessed by echocardiography. Inclusion criteria: Age ≥18 years old, consistent with Sepsis‐3 diagnosis of sepsis. Exclusion criteria: pregnant or lactating women; patients with mental illness, cognitive impairment, and unable to cooperate with evaluation; patients with advanced malignant tumors or end‐stage diseases with an expected survival period of <3 months; patients with chronic underlying diseases, congenital immune deficiencies; patients with heart disease, or who have received cardiopulmonary resuscitation.

Another 80 healthy volunteers who completed physical examinations in our hospital during the same period were selected as the healthy control group. This group of research subjects had no major organic diseases or other abnormal health conditions.

Venous blood was collected within 24 h after the patient was admitted to the intensive care unit, and the Acute Physiology and Chronic Health Evaluation II score and Sequential Organ Failure Assessment (SOFA)score were estimated.

### 2.2 Establishment of CLP rat models

Adult male Sprague‐Dawley (SD) rats weighing between 200 and 220 g were collected from the Shanghai Animal Center. The animals were maintained in a specific pathogen‐free environment with controlled temperature (23 ± 1°C), relative humidity (50 ± 5%), and a 12‐h light/dark cycle. All rats were allowed free access to food and water.

Sepsis was modeled by CLP as previously described.^39^ Rats were anesthetized with an intraperitoneal injection of 40 mg/kg sodium pentobarbital, shaved, and secured in the supine position. The surgical area was disinfected with 75% ethanol and povidone‐iodine. Sterilized instruments were used to make a 2–3 cm midline abdominal incision, and blunt dissection was performed to expose the cecum without compromising mesenteric blood flow. The distal one‐third of the cecum was ligated with 3‐0 silk sutures. An 18‐gauge (18G) needle was used to puncture the ligated segment of the cecum three times in a penetrating manner, while avoiding vascular injury. The cecum was then returned to the abdominal cavity, and the peritoneum and skin were sutured intermittently with 4‐0 silk sutures. Immediately post‐operatively, animals received 50 mL/kg sterile saline subcutaneously and were kept on a thermostatically controlled heating pad to prevent hypothermic shock. Sham‐operated controls underwent identical anesthesia and laparotomy without ligation or puncture.

In this study, SD rats were divided into several groups according to the intervention plan, with 10 rats in each group: the control group did not undergo CLP surgery and only received intraperitoneal injection of an equal volume of normal saline. The CLP group underwent CLP surgery to construct a sepsis model, and an equal volume of normal saline was injected intraperitoneally after surgery. MIR503HG negative control group, OE‐MIR503HG group, OE‐MIR503HG‐agomiR‐NC group, OE‐MIR503HG‐miR‐497‐5p‐agomiR group, OE‐MIR503HG‐miR‐497‐5p‐agomiR‐OE‐NC group, and the OE‐MIR503HG‐miR‐497‐5p‐agomiR‐OE‐BDNF group all underwent CLP surgery to construct a sepsis model, and were given corresponding interventions based on CLP treatment (corresponding vectors were injected intravenously 24 h before surgery, and the dose of injected vectors was 10 μg). To minimize selection bias, rats were randomly assigned to experimental groups using a computer‐generated randomization sequence. Furthermore, to reduce observation bias, all hemodynamic measurements, echocardiographic assessments, and histological analyses were performed by investigators who were blinded to the group allocation.

At the end of the animal experiment, all rats were euthanized by intraperitoneal injection of an overdose of sodium pentobarbital (150 mg/kg body weight),^40^ which was consistent with the guidelines of the American Veterinary Medical Association for the euthanasia of animals. This method ensures rapid and humane loss of consciousness and death, minimizing pain and distress. The dosage and route of administration were chosen based on previous studies and institutional animal care protocols to guarantee effective and ethical euthanasia.

All animal experiments were conducted in accordance with the guidelines for the care and use of laboratory animals and were approved by the Ruikang Hospital Affiliated to Guangxi University of Chinese Medicine Ethics Committee.

### 2.3 Quantitative reverse transcription polymerase chain reaction (RT‐qPCR)

Before RNA isolation, peripheral venous blood samples were collected and immediately centrifuged at 3000 rpm for 10 min at 4°C. The supernatant serum was transferred to RNase‐free tubes and stored at −80°C within 30 min of collection to avoid RNA degradation. Total RNA was isolated from serum using TRIzol LS reagent according to the manufacturer's instructions. RNA purity and concentration were evaluated using a NanoDrop‐2000 spectrophotometer. Only RNA samples with an A260/A280 ratio between 1.8 and 2.1 were deemed sufficiently pure and used for subsequent reverse transcription and qPCR analysis. Reverse transcription was performed using the PrimeScript RT Master Mix. RT‐qPCR was run on a StepOnePlus system with the miScript SYBR Green PCR Kit. Relative gene expression levels were calculated using the 2^−ΔΔCt^ method. For mRNA detection, glyceraldehyde‐3‐phosphate dehydrogenase (GAPDH) was used as the endogenous reference gene; for miRNA detection, U6 small nuclear RNA (U6) served as the internal control. The ΔCt value was calculated by subtracting the Ct value of the reference gene from the Ct value of the target gene. The ΔΔCt value was obtained by subtracting the ΔCt value of the control group from the ΔCt value of the experimental group. The final relative expression level was computed as 2^−ΔΔCt^.

### 2.4 Indicator measurement

To evaluate the cardiac function of model rats in each group, hemodynamic parameters such as left ventricular systolic pressure (LVSP), left ventricular end‐diastolic pressure (LVEDP), and left ventricular pressure maximum change rate (±dp/dt) were monitored and data collected through the MFLab 3.01 software using the FDP‐1 heart rate variability and baroreceptor sensitivity system.

The venous blood of rats in each group was collected, and the levels of cTnI and creatine kinase isoenzyme MB (CK‐MB), interleukin‐1 beta (IL‐1β), tumor necrosis factor‐alpha (TNF‐α), and interleukin‐6 (IL‐6) in the serum were measured by Enzyme‐linked immunosorbent assay to comprehensively evaluate the cardiac function and inflammatory response level of rats in each group.

### 2.5 RNA immunoprecipitation (RIP) assay

A pre‐cooled RIP lysis buffer was used for sample lysis, and the supernatant was collected after centrifugation. Protein A/G magnetic beads were incubated with the target protein antibody (or immunoglobulin G [IgG] control) to form complexes, which were then mixed with the lysate supernatant and incubated overnight for protein‐RNA complex capture. Following thorough washing to remove non‐specific binding, RNA was dissociated using proteinase K eluate and analyzed by RT‐qPCR to verify the enrichment of target RNA.

### 2.6 Dual luciferase assay

Wild‐type vectors (WT‐MIR503HG and WT‐BDNF) containing the miR‐497‐5p binding site, as well as their corresponding mutant vectors (MUT‐MIR503HG and MUT‐BDNF) with disrupted binding sites, were constructed. Logarithmically growing 293T cells were seeded into 96‐well plates; at 70%–80% confluence, cells were grouped for co‐transfection with wild‐type/mutant vectors and miR‐497‐5p‐mimics/inhibitors. Following 48 h of culture, luciferase activity was detected, and relative activity was normalized to Renilla luciferase as the internal control.

### 2.7 KEGG analysis

Potential target genes downstream of miR‐497‐5p were obtained through the intersection screening of three online databases: ENCORI, TargetScan, and miRDB. Kyoto Encyclopedia of Genes and Genomes (KEGG) analysis was used to predict the signaling pathways that may be related to the intersection of target genes.

### 2.8 Statistical analysis

All analyses were performed with SPSS 21.0. Data were presented as mean ± standard deviation (SD) from at least five independent experiments or biological replicates. Receiver operating characteristic (ROC) curves were constructed to assess the diagnostic performance of MIR503HG for sepsis and SICD. The Pearson correlation coefficient was used to analyze the relationships between target genes as well as the relationships between target genes and clinical indicators. Categorical variables were compared using the Chi‐square (*χ* ^2^) test. For comparisons between two groups, an unpaired two‐tailed Student's *t*‐test was applied. For comparisons involving three or more groups, one‐way analysis of variance was employed, followed by Tukey's post‐hoc test for multiple comparisons when equal variances were assumed. A *p* < 0.05 was considered statistically significant.

## 3 RESULTS

### 3.1 General clinical information of the participants' comparison

This study compared the clinical characteristics of 80 controls and 102 sepsis patients, and further divided the sepsis patients into the SICD group (*n* = 31) or non‐SICD group (*n* = 71). The results showed that the levels of cTnI, CK‐MB, and inflammatory factors in the total sepsis group were significantly higher than those in the control group, and the above indicators in the SICD group were significantly higher than those in the non‐SICD group. In addition, the LVEF of the total sepsis group was lower than that of the control group, and the LVEF of the SICD group was much lower than that of the non‐SICD group. In addition, the SOFA score and APACHE II score in the SICD group were significantly higher than those in the non‐SICD group. The above results suggested that sepsis could induce myocardial damage and cardiac function decline, and that SICD patients have more severe inflammatory reactions and more critical conditions (Table 1).

**TABLE 1:** The general clinical characteristics of the participants.

Characteristics	Control (*n* = 80)	Sepsis total (*n* = 102)	*p*‐value	Sepsis (*n* = 102)		*p*‐value
				No cardiac dysfunction (*n* = 71)	Cardiac dysfunction (*n* = 31)	
Age (years)	55.83 ± 5.34	56.36 ± 4.40	0.463	56.31 ± 4.44	56.45 ± 4.40	0.882
Sex (male/female)	43/37	61/41	0.413	37/34	18/13	0.579
BMI (kg/m^2^)	21.47 ± 1.89	22.01 ± 1.90	0.061	22.00 ± 1.85	22.01 ± 2.05	0.987
Smoking (YES/NO)	24/56	42/60	0.120	18/53	10/21	0.472
cTnI (ug/L)	0.10 ± 0.05	0.90 ± 0.78	<0.001	0.54 ± 0.63	1.71 ± 0.39	<0.001
CK‐MB (U/L)	14.67 ± 3.31	33.13 ± 7.55	<0.001	30.08 ± 6.51	40.13 ± 4.48	<0.001
LVEF (%)	61.97 ± 7.24	52.55 ± 6.91	<0.001	55.99 ± 4.68	44.68 ± 4.21	<0.001
IL‐6 (ng/L)	11.16 ± 2.72	89.28 ± 13.79	<0.001	83.16 ± 11.07	103.30 ± 7.90	<0.001
TNF‐α (ng/L)	19.75 ± 4.11	122.05 ± 39.28	<0.001	104.64 ± 30.35	161.90 ± 26.18	<0.001
SOFA score		5.71 ± 1.85		4.87 ± 1.39	7.63 ± 1.21	<0.001
APACHE II score		19.50 ± 4.71		17.87 ± 4.49	23.22 ± 2.66	<0.001

Abbreviations: APACHE II score, Acute Physiology and Chronic Health Evaluation II score; BMI, Body Mass Index; CK‐MB, Creatine Kinase‐MB; cTnI, Cardiac Troponin I; IL‐6, Interleukin‐6; LVEF, Left Ventricular Ejection Fraction; SOFA score, Sequential Organ Failure Assessment score; TNF‐α, Tumor Necrosis Factor‐α.

### 3.2 Expression of MIR503HG in sepsis and SICD

**FIGURE 1:**
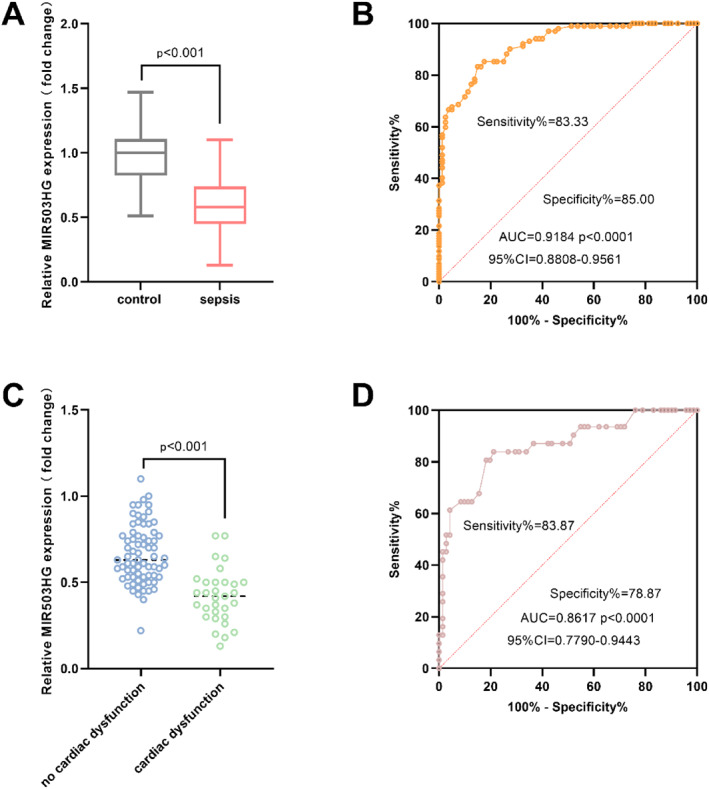
Expression of MIR503HG in sepsis and SICD. (A). MIR503HG expression was significantly reduced in patients with sepsis. (B). MIR503HG exhibited strong diagnostic performance for distinguishing sepsis from controls. (C). The relative expression of MIR503HG in SICD was markedly lower than in those without cardiac complications. (D). MIR503HG also demonstrated considerable diagnostic potential for identifying SICD. Data were presented as mean ± standard deviation. Comparisons between two groups were performed using unpaired two‐tailed Student's *t*‐test. Receiver operating characteristic curves were generated and the area under the curve values calculated. *p* < 0.05 was considered statistically significant. SICD, sepsis‐induced cardiac dysfunction.

In the sepsis group, the MIR503HG expression was significantly down‐regulated (Figure 1A). Its ROC curve for diagnosing sepsis showed that the area under the curve (AUC) reached 0.9184 (95% CI: 0.8808–0.9561, *p* < 0.0001), corresponding to a sensitivity of 83.33% and a specificity of 85.00%, indicating that MIR503HG has good diagnostic differentiation performance for sepsis (Figure 1B). MIR503HG expression was markedly lower in SICD patients than in septic individuals without cardiac compromise, indicating that reduced expression was linked to SICD (Figure 1C). ROC analysis yielded an AUC of 0.8617 (95% CI: 0.7790–0.9443, *p* < 0.0001), with 83.87% sensitivity and 78.87% specificity, underscoring the utility of MIR503HG for identifying SICD (Figure 1D). Collectively, these data showed that MIR503HG was down‐regulated in sepsis—especially when accompanied by cardiac dysfunction—and may serve as a reliable biomarker for both sepsis and SICD.

Furthermore, the correlation analysis revealed that the MIR503HG expression was significantly negatively correlated with myocardial injury markers (cTnI, CK‐MB), cardiac function indicators (LVEF), inflammatory factors (IL‐6, TNF‐α), and severity scores of the disease (SOFA, APACHE II) (Table 2).

**TABLE 2:** The correlation between MIR503HG expression and clinical indicators.

Characteristics	*R*	*p*‐value
cTnI (ug/L)	−0.682	<0.001
CK‐MB (U/L)	−0.722	<0.001
LVEF (%)	−0.765	<0.001
IL‐6 (ng/L)	−0.754	<0.001
TNF‐α (ng/L)	−0.731	<0.001
SOFA score	−0.738	<0.001
APACHE II score	−0.706	<0.001

Abbreviations: APACHE II score, Acute Physiology and Chronic Health Evaluation II score; CK‐MB, Creatine Kinase‐MB; cTnI, Cardiac Troponin I; IL‐6, Interleukin‐6; LVEF, Left Ventricular Ejection Fraction; SOFA score, Sequential Organ Failure Assessment score; TNF‐α, Tumor Necrosis Factor‐α.

### 3.3 The role of MIR503HG in the animal model of sepsis with CLP

**FIGURE 2:**
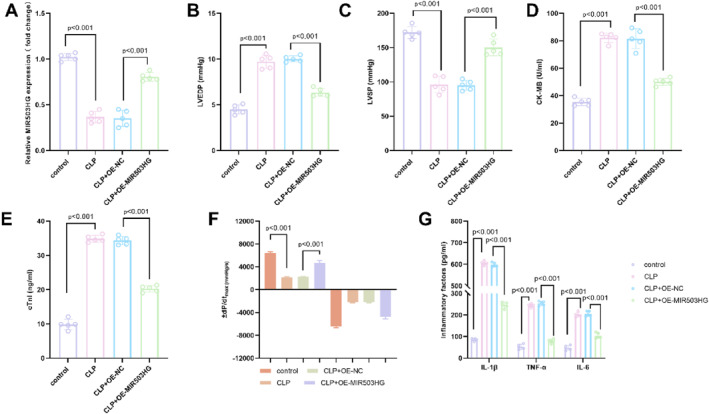
Functional role of MIR503HG in a rat CLP model of sepsis. (A). In the CLP model, MIR503HG expression was significantly downregulated. However, this deficiency was effectively restored by MIR503HG overexpression, confirming the efficacy of the intervention strategy. (B–F). CLP induced significant cardiac injury, characterized by elevated left ventricular end‐diastolic pressure (B), reduced left ventricular systolic pressure (C), increased levels of myocardial injury biomarkers—CK‐MB and cTnI—(D, E), and impaired myocardial contractility and relaxation, as reflected by altered ± dP/dt maxima (F). Notably, overexpression of MIR503HG ameliorated all these pathological changes. (G). The sepsis‐induced upregulation of pro‐inflammatory cytokines—including IL‐1β, tumor necrosis factor‐alpha, and IL‐6—was substantially suppressed following MIR503HG overexpression. Data were presented as mean ± standard deviation. Comparisons among multiple groups were performed using one‐way analysis of variance followed by Tukey's post‐hoc test. *p* < 0.05 was considered statistically significant.

In the CLP model, the MIR503HG expression was significantly down‐regulated. However, after intervention with overexpression of MIR503HG, its expression increased significantly, successfully verifying the effectiveness of the overexpression system (Figure 2A). CLP induced significant cardiac dysfunction, characterized by elevated LVEDP (Figure 2B) and decreased LVSP (Figure 2C). Concurrently, marked upregulation of myocardial injury markers, including CK‐MB and cTnI, was observed (Figure 2D,E), along with evident impairment of ±dP/dt, the core indices of myocardial systolic and diastolic function (Figure 2F). Notably, overexpression of MIR503HG reversed these abnormalities: LVEDP decreased, LVSP increased, CK‐MB and cTnI levels were downregulated, and ±dP/dt was restored. Moreover, the CLP‐driven surge of pro‐inflammatory cytokines—IL‐1β, TNF‐α, and IL‐6—was markedly blunted after MIR503HG overexpression (Figure 2G). Taken together, these findings established MIR503HG as a protective player in sepsis: its up‐regulation effectively mitigated cardiac injury and dampened the systemic inflammatory response.

### 3.4 MIR503HG may exert its effect by regulating the miR‐497‐5p/BDNF axis

**FIGURE 3:**
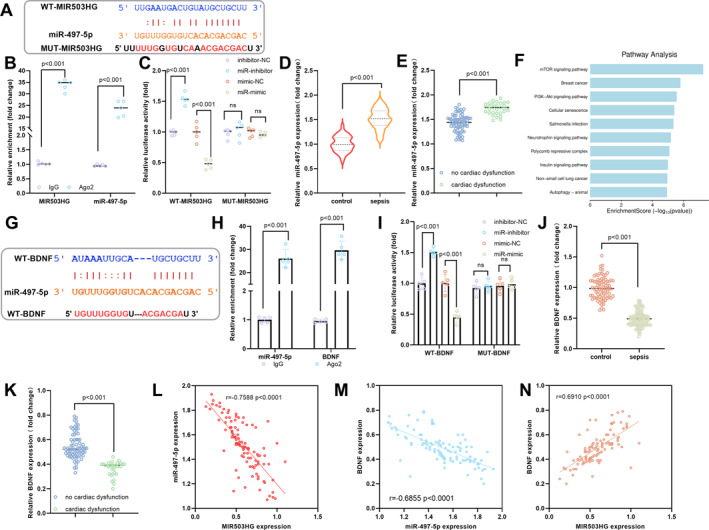
MIR503HG regulated the miR‐497‐5p/BDNF axis. (A). Bioinformatic analysis revealed complementary binding sites between WT‐MIR503HG and miR‐497‐5p. (B). RIP assays provided preliminary evidence that MIR503HG and miR‐497‐5p were co‐enriched in Ago2‐containing complexes, suggesting direct interaction. (C). Dual‐luciferase reporter assays confirmed that MIR503HG directly targeted miR‐497‐5p. (D). Clinically, miR‐497‐5p expression was significantly elevated in septic patients compared to healthy controls. (E). Moreover, miR‐497‐5p levels were higher in and SICD patients. (F). Kyoto Encyclopedia of Genes and Genomes pathway enrichment analysis indicated that the potential downstream target genes of miR‐497‐5p were involved in key signaling pathways such as mechanistic target of rapamycin and neurotrophin signaling. (G). The 3′ untranslated region (3′UTR) of BDNF contains conserved sequences complementary to miR‐497‐5p. (H). RIP assays further showed that both miR‐497‐5p and BDNF were enriched in Ago2 immunoprecipitates, supporting their functional interaction within the RNA‐induced silencing complex complex. (I). Luciferase assays validated direct targeting of BDNF by miR‐497‐5p. (J). BDNF expression was significantly downregulated in the septic patients. (K). Additionally, BDNF levels were lower in SICD patients. (L–N). Correlation analyses revealed a negative correlation between MIR503HG and miR‐497‐5p (L), a negative correlation between miR‐497‐5p and BDNF (M), and a positive correlation between MIR503HG and BDNF (N). ns indicates no statistical significance. Data were presented as mean ± standard deviation. Two‐group comparisons were performed using unpaired two‐tailed Student's *t*‐test. For multiple group comparisons, one‐way analysis of variance with Tukey's post‐hoc test was applied. Pearson correlation coefficient was used for correlation analyses. *p* < 0.05 was considered statistically significant. BDNF, brain‐derived neurotrophic factor; RIP, RNA immunoprecipitation; SICD, sepsis‐induced cardiac dysfunction.

The sequence of WT‐MIR503HG has a complementary binding region with miR‐497‐5p (Figure 3A). RIP experiment results showed that both MIR503HG and miR‐497‐5p were significantly enriched in the Argonaute 2 (Ago2) precipitation group, suggesting that they could bind and enter the RNA‐induced silencing complex (RISC), preliminarily confirming their potential target binding relationship (Figure 3B). The luciferase experiment further clarified: when WT‐MIR503HG was co‐transfected with miR‐497‐5p‐mimic, the luciferase activity was significantly reduced. While MUT‐MIR503HG with a mutated binding site had no such effect, it directly verified the targeted binding relationship between MIR503HG and miR‐497‐5p (Figure 3C). In the clinical samples, the miR‐497‐5p expression was significantly increased in sepsis patients (Figure 3D), and the miR‐497‐5p expression in the SICD patients was higher than that in the sepsis subgroup without cardiac dysfunction (Figure 3E). Bioinformatics enrichment analysis showed that miR‐497‐5p was mainly involved in the mechanistic target of rapamycin signaling pathway, neurotrophic factor signaling pathway, etc., suggesting that it played a role in core biological processes such as cell proliferation and survival (Figure 3F). B A complementary binding sequence between miR‐497‐5p and the 3′ untranslated region (3′UTR) of BDNF was identified (Figure 3G). RIP assays confirmed that both miR‐497‐5p and BDNF were significantly enriched in the Ago2‐precipitated fraction, indicating that they could bind to each other and assemble into the RISC (Figure 3H). Dual‐luciferase reporter assays further validated this interaction: co‐transfection of WT‐BDNF with miR‐497‐5p‐mimic resulted in a marked reduction in luciferase activity, whereas no such effect was observed in the group transfected with MUT‐BDNF (carrying mutations at the binding site). These findings definitively established the direct targeted binding relationship between miR‐497‐5p and BDNF (Figure 3I). In patients with sepsis, the BDNF expression was significantly decreased (Figure 3J), and BDNF expression in the SICD patients was lower than that in the sepsis subgroup without cardiac dysfunction (Figure 3K). Correlation analysis results showed that MIR503HG was negatively correlated with the miR‐497‐5p expression (*r* = −0.7588, Figure 3L). miR‐497‐5p was negatively correlated with the BDNF expression (*r* = −0.6855, Figure 3M), whereas MIR503HG was positively correlated with BDNF expression (*r* = 0.6910, Figure 3N).

### 3.5 MIR503HG regulated SICD and inflammation through the miR‐497‐5p/BDNF axis

**FIGURE 4:**
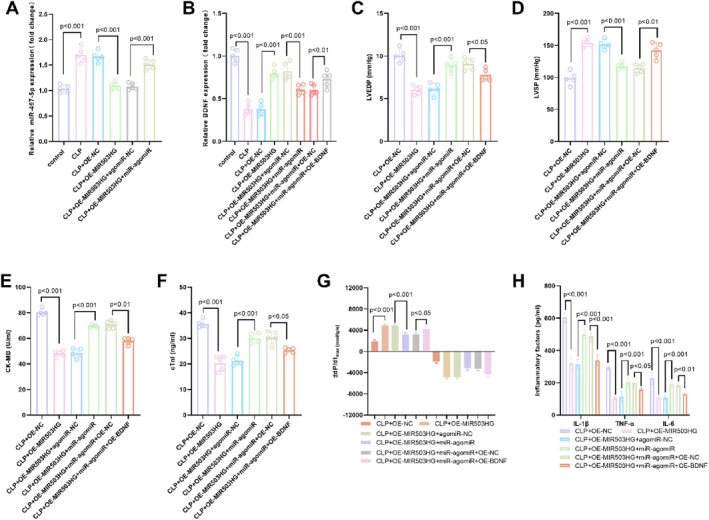
MIR503HG alleviated SICD and inflammation via the miR‐497‐5p/BDNF axis. (A). In the CLP model, MIR503HG overexpression significantly reduced miR‐497‐5p expression. Subsequent administration of miR‐497‐5p agomiR successfully reversed this suppression, validating the specificity of miR‐497‐5p modulation. (B). MIR503HG overexpression enhanced BDNF expression, whereas miR‐497‐5p agomiR attenuated this effect. Importantly, exogenous BDNF overexpression restored BDNF levels, confirming effective intervention at this node. (C–H). Rescue experiments demonstrated that miR‐497‐5p agomiR partially abrogated the cardioprotective effects of MIR503HG: re‐elevation of LVEDP (C), reduction in LVSP (D), increased CK‐MB and cTnI levels (E, F), worsened ± dP/dt values (G), and heightened inflammatory cytokine production (IL‐1β, TNF‐α, IL‐6) (H) were observed upon miR‐497‐5p restoration. Conversely, BDNF overexpression counteracted these detrimental effects—reducing LVEDP, increasing LVSP, lowering injury markers, improving contractile function, and suppressing inflammation—thereby confirming that BDNF acted downstream of miR‐497‐5p to mediate MIR503HG's protective role in SICD and systemic inflammation. Data were presented as mean ± SD. Comparisons among multiple groups were performed using one‐way ANOVA followed by Tukey's post‐hoc test. *p* < 0.05 was considered statistically significant. ANOVA, analysis of variance; BDNF, brain‐derived neurotrophic factor; CK‐MB, creatine kinase‐MB; cTnI, cardiac troponin I; LVEDP, left ventricular end‐diastolic pressure; SICD, sepsis‐induced cardiac dysfunction; LVSP, left ventricular systolic pressure; SD, standard deviation; TNF‐α, tumor necrosis factor‐alpha.

In the CLP sepsis model, MIR503HG over‐expression markedly repressed miR‐497‐5p levels; subsequent delivery of miR‐497‐5p‐agomiR partly restored its abundance, confirming efficient on‐target modulation (Figure 4A). Likewise, MIR503HG up‐regulation significantly elevated BDNF expression, an effect reversed by co‐administration of miR‐497‐5p‐agomiR. Re‐introduction of BDNF rescued its expression levels, validating the efficacy of the BDNF intervention (Figure 4B). Functional rescue assays confirmed that miR‐497‐5p agomiR could partially reverse the protective effects of MIR503HG. Specifically, overexpression of miR‐497‐5p exacerbated the myocardial diastolic (increased LVEDP, Figure 4C) and systolic (decreased LVSP, Figure 4D) dysfunction that had been ameliorated by MIR503HG in the CLP model. Meanwhile, it led to elevated levels of myocardial injury markers (CK‐MB and cTnI, Figure 4E,F), impaired myocardial systolic/diastolic function (±dP/dt, Figure 4G), and increased production of pro‐inflammatory cytokines (Figure 4H). But BDNF could restore the effect of MIR503HG: Overexpression of BDNF could once again reduce LVEDP, increase LVSP, cause CK‐MB and cTnI to decrease, improve ± dP/dt, and lower the levels of inflammatory factors.

## 4 DISCUSSION

SICD is a common complication in patients with sepsis and an important cause of death in patients with sepsis.^41^, ^42^ MIR503HG plays a tumor suppressor role in a variety of cancers and is involved in regulating the inflammatory response of the disease.^24^, ^43^, ^44^ However, its underlying molecular mechanisms in sepsis and SICD remain unclear. By integrating clinical data analysis and constructing CLP animal models, this study discovered for the first time that MIR503HG was significantly down‐regulated in sepsis, especially in patients with cardiac dysfunction. In sepsis mouse models, overexpression of MIR503HG could effectively reduce cardiac function damage, reduce myocardial injury markers, and inhibit systemic inflammatory responses. Moreover, MIR503HG may act as a ceRNA by adsorbing miR‐497‐5p, releasing its inhibition on BDNF, and ultimately exerting a cardioprotective effect in sepsis.

SICD is an important factor contributing to poor patient prognosis.^45^ Through integrating clinical data, we found that the levels of myocardial injury markers (cTnI, CK‐MB) and inflammatory factors in the total sepsis group were significantly higher than those in the control group. Moreover, in the subgroup with combined cardiac dysfunction, these indicators further increased, accompanied by a significant decrease in LVEF. This suggested that the intensification of the inflammatory response and the aggravation of myocardial injury were the core pathological basis of sepsis. The SOFA and APACHE II scores in the SICD group were significantly higher, indicating that myocardial injury and decreased cardiac function might be important factors driving the progression of sepsis. In addition, MIR503HG is down‐regulated in a variety of tumors and participates in inflammatory responses.^26^, ^46^, ^47^ We found that MIR503HG was significantly underexpressed in patients with sepsis, and its expression was lowest in SICD patients. It could not only be used as a diagnostic marker for sepsis, but also effectively identify whether sepsis is complicated by cardiac dysfunction. More importantly, the MIR503HG expression was negatively correlated with cTnI, CK‐MB, and inflammatory factors in clinical indicators, positively correlated with LVEF, and negatively correlated with SOFA and APACHE II scores. This indicated that low expression of MIR503HG may reflect disease severity, myocardial damage, and cardiac function deterioration, and may identify patients at high risk for cardiac dysfunction in sepsis at an early stage.

To rigorously define the role of MIR503HG, we employed the CLP rats model of sepsis. In CLP mice, MIR503HG expression fell in parallel with contractile dysfunction (elevated LVEDP, reduced LVSP, impaired ± dP/dt), rising serum myocardial injury biomarkers, and a surge of pro‐inflammatory cytokines. Restoring MIR503HG levels partly reversed these abnormalities: it rescued both systolic and diastolic performance, blunted cardiomyocyte injury, and subdued systemic inflammation. These data established MIR503HG as a dual‐action guardian that simultaneously protects the myocardium and restrains inflammation during sepsis.

One of the core mechanisms by which lncRNA plays a biological regulatory role is to adsorb miRNAs through the “molecular sponge” effect, thereby competitively inhibiting the binding of miRNAs to their target genes, thereby regulating the expression of downstream genes.^48^ Mechanistically, we used RIP and dual‐luciferase reporter genes to jointly confirm the targeted binding of MIR503HG to miR‐497‐5p and the targeted binding of miR‐497‐5p to BDNF. In clinical samples and CLP rat models, we observed that the expression of MIR503HG and BDNF was down‐regulated during sepsis, whereas the miR‐497‐5p expression was up‐regulated, and there was a significant correlation between the expression levels of the three (MIR503HG was negatively correlated with miR‐497‐5p and positively correlated with BDNF, miR‐497‐5p was negatively correlated with BDNF). This is also consistent with previous studies. Previous research has shown that down‐regulation of miR‐497‐5p improves acute lung injury induced by sepsis,^31^ overexpression of miR‐497‐5p can promote apoptosis and inflammation in LPS‐induced BEAS‐2B cells,^30^ and up‐regulation of BDNF can significantly inhibit the inflammatory response in mice induced by CLP.^37^ We also found that on the basis of overexpressing MIR503HG, additional supplementation of miR‐497‐5p‐agomiR could partially offset the improvement of cardiac function, reduction of myocardial damage, and anti‐inflammatory effects brought by MIR503HG. On this basis, overexpression of BDNF could restore the protective effect. Based on the classic ceRNA theory, we propose the following action model: when MIR503HG was highly expressed, MIR503HG acted as a “molecular sponge” to absorb a large amount of miR‐497‐5p, thereby weakening the inhibition of miR‐497‐5p on the downstream target gene BDNF mRNA and allowing BDNF to be expressed normally. BDNF, a key neurotrophin, has been shown to exert broad cardioprotective actions: it enhances cardiomyocyte survival, suppresses apoptosis, improves energy metabolism, and counteracts cardiac fibrosis.^49^, ^50^ In the state of sepsis, the MIR503HG expression decreased sharply, resulting in the weakening of its “sponge” adsorption capacity, and the level of free miR‐497‐5p increases, which in turn strengthens the inhibition of BDNF, ultimately leading to insufficient expression of BDNF. The weakening of BDNF protective signals may induce or aggravate cardiac dysfunction.

This study was the first to link MIR503HG to sepsis and SICD, and through in vitro experiments, it has been confirmed that its molecular pathway regulates the miR‐497‐5p/BDNF axis through the ceRNA mechanism to exert cardioprotective effects. However, this study also has some limitations. First, the clinical sample size was relatively modest (*n* = 102 sepsis patients, including 31 with SICD), and patient recruitment was conducted at a single center. The limited sample size may reduce statistical power to detect smaller effect sizes, and the single‐center design may restrict the generalizability of our findings to broader, more ethnically and geographically diverse patient populations. Future multi‐center prospective cohort studies with larger sample sizes were essential to validate the diagnostic and prognostic utility of MIR503HG across different clinical settings and to establish standardized cut‐off values for clinical application. Second, this study relied exclusively on clinical sample analyses and in vivo experiments using the CLP rat model, without complementary in vitro cellular mechanistic validation. Consequently, the direct cell‐autonomous effects of MIR503HG on cardiomyocyte survival, apoptosis, and inflammatory signaling cannot be definitively established. It remains possible that the observed cardioprotective and anti‐inflammatory benefits are mediated, at least in part, through indirect systemic or immune cell‐dependent mechanisms. Future studies employing cultured cardiomyocyte cell lines (e.g., H9c2 cells or primary neonatal rat ventricular cardiomyocytes) under septic or inflammatory stress conditions are warranted to delineate the precise intracellular pathways through which the MIR503HG/miR‐497‐5p/BDNF axis exerts its protective functions. Third, although we focused on the miR‐497‐5p/BDNF axis, we cannot exclude the possibility that MIR503HG functions through additional ceRNA networks or alternative molecular mechanisms, such as direct protein binding or epigenetic regulation. Comprehensive transcriptomic and proteomic analyses will be required to fully map the MIR503HG interactome in the context of sepsis and SICD. Fourth, the potential translational application of MIR503HG as a therapeutic target warrants exploration; future studies should investigate whether targeted delivery of MIR503HG mimics or small molecules that enhance its expression can confer clinical benefit in septic patients. Despite these limitations, we believe our findings provide a robust and compelling foundation for future investigations into the diagnostic and therapeutic potential of targeting the MIR503HG/miR‐497‐5p/BDNF axis in sepsis SICD.

## 5 CONCLUSION

The MIR503HG expression was downregulated in sepsis patients and SICD patients, as well as in the CLP rat model. The downregulation of its expression leads to the release of inhibition on miR‐497‐5p, thereby suppressing the BDNF expression, ultimately exacerbating myocardial injury, impairing cardiac function, and amplifying the inflammatory response.

## AUTHOR CONTRIBUTIONS

**Pingping Qi**: Conceptualization; data curation; formal analysis; resources; methodology; writing—original draft. **Minghe Jiang**: Formal analysis; investigation. **Minsheng Wu**: Formal analysis; investigation. **Shengkui Chen**: Formal analysis; investigation. **Qile Ye**: Data curation; writing—review and editing. **Rong Tang**: Conceptualization; formal analysis; funding acquisition; methodology; resources; writing—original draft; writing—review and editing.

## CONFLICT OF INTEREST STATEMENT

The authors have declared no conflict of interest.

## ETHICS STATEMENT

This study was approved by the Ruikang Hospital Affiliated to Guangxi University of Chinese Medicine Ethics Committee, and all participants signed the informed consent form. All animal experiments were conducted in accordance with the guidelines for the care and use of laboratory animals and were approved by the Ruikang Hospital Affiliated to Guangxi University of Chinese Medicine Ethics Committee.

## Data Availability

The data that support the findings of this study are available on request from the corresponding author.
